# Thiol trapping and metabolic redistribution of sulfur metabolites enable cells to overcome cysteine overload

**DOI:** 10.15698/mic2017.04.567

**Published:** 2017-03-27

**Authors:** Anup Arunrao Deshpande, Muskan Bhatia, Sunil Laxman, Anand Kumar Bachhawat

**Affiliations:** 1 Department of Biological Sciences, Indian Institute of Science Education and Research (IISER Mohali), S.A.S. Nagar, Punjab 140306, India.; 2 Institute for Stem Cell Biology and Regenerative Medicine (inStem), NCBS campus, Bangalore 560065, India.

**Keywords:** cysteine, toxicity, thiol, polyamine, biosynthesis, Saccharomyces cerevisiae

## Abstract

Cysteine is an essential requirement in living organisms. However, due to its reactive thiol side chain, elevated levels of intracellular cysteine can be toxic and therefore need to be rapidly eliminated from the cellular milieu. In mammals and many other organisms, excess cysteine is believed to be primarily eliminated by the cysteine dioxygenase dependent oxidative degradation of cysteine, followed by the removal of the oxidative products. However, other mechanisms of tackling excess cysteine are also likely to exist, but have not thus far been explored. In this study, we use *Saccharomyces cerevisiae*, which naturally lacks a cysteine dioxygenase, to investigate mechanisms for tackling cysteine overload. Overexpressing the high affinity cysteine transporter, *YCT1*, enabled yeast cells to rapidly accumulate high levels of intracellular cysteine. Using targeted metabolite analysis, we observe that cysteine is initially rapidly interconverted to non-reactive cystine *in vivo*. A time course revealed that cells systematically convert excess cysteine to inert thiol forms; initially to cystine, and subsequently to cystathionine, S-Adenosyl-L-homocysteine (SAH) and S-Adenosyl L-methionine (SAM), in addition to eventually accumulating glutathione (GSH) and polyamines. Microarray based gene expression studies revealed the upregulation of arginine/ornithine biosynthesis a few hours after the cysteine overload, and suggest that the non-toxic, non-reactive thiol based metabolic products are eventually utilized for amino acid and polyamine biogenesis, thereby enabling cell growth. Thus, cells can handle potentially toxic amounts of cysteine by a combination of thiol trapping, metabolic redistribution to non-reactive thiols and subsequent consumption for anabolism.

## INTRODUCTION

Cysteine is essential for the survival of all cells, and is obtained either through *de novo *biosynthesis, or is transported into cells from the extracellular medium by amino acid transporters. In organisms such as bacteria, archaea, fungi and plants, cysteine biosynthesis occurs *de novo *from inorganic sulfate 1,2,3,4. In mammals, cysteine biosynthesis from inorganic sulfate is absent, but cysteine is synthesized from methionine via the trans-sulfuration pathway, and is also obtained directly from nutritional sources 5,6. Cysteine transporters have been described across phyla 7,8,9,10. Contrastingly, cystine transporters, though not as ubiquitous, are also prevalent in different organisms 11,12,13,14,15.

Due to its highly reactive thiol group, sudden, high concentrations of cysteine can be harmful to the cell. Indeed, cysteine toxicity has been reported in bacteria, yeasts and fungi 16,17,18. In mammals, high cysteine levels are extremely toxic especially in neuronal cells 19,20. Compared to other amino acids, the intracellular cysteine concentrations in the cell appear to be maintained at much lower levels at steady state 21, and may therefore be a limiting amino acid for many processes.

Cysteine homeostasis, therefore, is maintained through tight regulatory control of biosynthesis and transport in microbial cells 1,2,3,4. In mammalian cells, while the regulation of cysteine homeostasis is less studied, it is thought to be primarily maintained by the removal of excess cysteine through its degradation by cysteine dioxygenase 5. Cysteine dioxygenase converts cysteine to cysteine-sulfinate, which in turn is utilized for the generation of hypotaurine/taurine, sulfite, sulfate, pyruvate and H_2_S 5. In the pathogenic yeast *C. albicans*, cysteine dioxygenase (*CDG1*) can degrade excess cysteine, and the sulfite generated is effluxed out through the sulfite efflux pump, *SSU1 *18. However, despite the proposed central role of cysteine dioxygenase in cysteine homeostasis, a knockout of cysteine dioxygenase in mice surprisingly led to only a small increase in intracellular cysteine concentrations. Here, in the absence of cysteine dioxygenease, the non-oxidative desulfuration pathway was hypothesized to play a role in cysteine detoxification 22. Collectively, these data suggest the existence of other mechanisms to handle cysteine overload, which remain uninvestigated.

The budding yeast, *S. cerevisiae, *naturally lacks a cysteine dioxygenase and thus appears to be an excellent organism to evaluate alternate modes of handling cysteine overload. Prior studies of cysteine and homocysteine toxicity in *S. cerevisiae *showed that yeast experienced an ER stress due to high intracellular concentrations of cysteine or homocysteine 17. However, the mechanisms by which *S. cerevisiae *handled cysteine overload remain unknown. Furthermore, this and earlier models have required very high concentrations of extracellular cysteine in order to observe and investigate toxicity. This is partly because although *S. cerevisiae* has multiple transporters that can transport cysteine, these are all of low affinity and specificity. The primary high affinity cysteine transporter-*YCT1* is strongly repressed by cysteine in the medium 7. Moreover, high levels of these amino acids in the medium can interfere with transport of other amino acids and other aspects of metabolism, and so the effects seen may be non-specific. We have therefore engineered and utilized a simple system where the specific cysteine transporter, *YCT1*, is constitutively expressed under a strong promoter in order to generate high cysteine levels in the cell, while adding relatively lower levels of the amino acid in the medium. Furthermore, we also employed a similarly expressed high affinity cystine transporter (*CgCYN1*) 12. To understand cellular responses in yeast cells to high levels of intracellular cysteine we adopted a two-pronged approach. We used a targeted, quantitative metabolite analysis in cells exposed to cysteine/cystine at different time intervals where the metabolites in the sulfur assimilation and metabolic pathway were estimated. We combined this with genome-wide expression profiling of the cells exposed to high levels of either intracellular cysteine or cystine, to understand genetic responses in adaptation to cysteine overload. Our results show that cysteine and cystine can rapidly interconvert *in vivo*, and that a principal mechanism of removal of excess cysteine is through its conversion into more inert forms of thiols, effectively a form of ‘thiol trapping’. Subsequently, cells can utilize these less-reactive thiols for multiple biosynthetic processes. These results reveal general ways of handling excess cysteine in the cell, and an elegant orchestration of metabolic processes to safely sequester and utilize cysteine.

## RESULTS

### Overexpression of the cysteine or cystine transporters leads to hypersensitivity of *S. cerevisiae* to cysteine or cystine

To determine if the constitutively expressed high affinity cysteine transporter (*YCT1*) or the heterologous *C. glabrata *cystine transporter (*CgCYN1*) in *S. cerevisiae* conferred sensitivity to these amino acids at lower media concentrations, *S. cerevisiae* cells were transformed with *YCT1* or *CgCYN1,* expressed under the strong constitutive TEF promoter, and analyzed on minimal SD media with different concentrations of cysteine or cystine.

**Figure 1:**
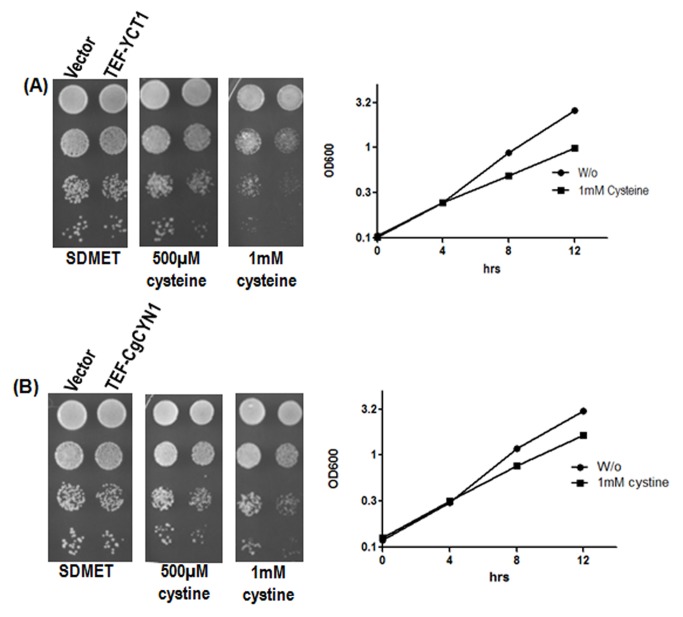
FIGURE 1: Effect of (A) cysteine and (B) cystine on *S. cerevisiae *cells expressing TEF-*YCT1* or TEF-*CgCYN1.* *S. cerevisiae* cysteine transporter *YCT1 *and *C. glabrata *cystine transporter *CgCYN1* expressed under TEF promoter and corresponding vector (p416TEF) were transformed into *S. cerevisiae *ABC 1738. Transformants were evaluated for growth and toxicity by serial dilution on minimal media containing different cysteine and cystine concentrations and growth curve analysis was carried in SD (synthetic defined) medium where 1 mM cysteine or cystine was added after 3 hrs of incubation at 30°C.

The growth comparisons done on plates with increasing concentrations of cysteine or cystine suggested that cysteine or cystine were not toxic to the yeasts up to 0.5 mM concentration. However, beginning at 0.5 mM, we observed retarded growth and increased toxicity both on plates and in liquid broth (Fig. 1). Thus, toxicity to cysteine was observed at substantially lower concentrations than previous report 17. We also confirmed these results in liquid broth with cysteine or cystine at concentrations of 0.5 and 1 mM (Fig. 1). These results suggest that these transporters allow the accumulation of these metabolites and thus did not require the high levels of cysteine in the medium to induce toxicity (as reported earlier) 17. We have used this concentration (0.5 mM) where toxicity becomes observable in our subsequent experiments.

### Metabolite analysis reveals a rapid inter-conversion of cysteine to cystine during cysteine overload

To understand how cells deal with an acute, large influx of either cysteine or cystine, we directly estimated the relative amounts of different metabolites in the sulfur related pathways over time. Beginning with 2.5 min after addition of cysteine, metabolites were extracted at 15, 60 and 300 min time intervals, and analyzed by targeted, quantitative LC-MS/MS. For comparative purposes, we looked at each individual metabolite under these different conditions.

**Figure 2:**
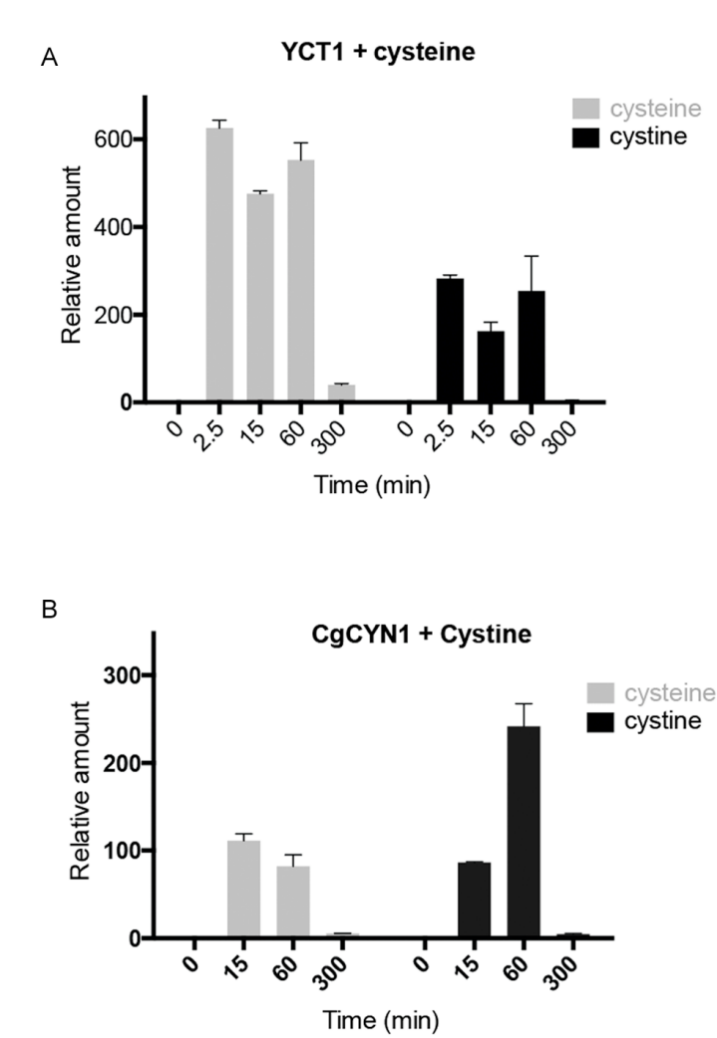
FIGURE 2: Relative intracellular amounts of cysteine and cystine as a function of time in cells overexpressing either cysteine transporter and challenged with cysteine or cystine transporter and challenged with cystine. **(A)** Relative levels of cysteine and cystine in *S. cerevisiae *cells expressing TEF-*YCT1* treated with 500 µM cysteine measured at different time points. **(B)** Relative levels of cysteine and cystine in *S. cerevisiae *cells expressing TEF-*CgCYN1* treated with 500 µM cystine measured at different time points.

In the *YCT1* expressing cells exposed to cysteine (Fig. 2A), an immediate, dramatic increase in intracellular cysteine amounts is observed. However, the level of cysteine drops after the first hour. Since the normal concentrations of cysteine in yeast cells grown in SD medium are in the range of 0.1-0.4 µM 21 under conditions where excess cysteine is pumped into the cells, the intracellular concentrations achieved were in the range of 60-240 µM. The intracellular cysteine concentrations eventually returned to initial, basal levels of the untreated conditions. Interestingly, in these same cells, we also observed high amounts of intracellular cystine (Fig. 2A). Since *YCT1* is known not to transport cystine 7, these data suggest that the cysteine transported inside the cells is converted to cystine.

*CgCYN1* expressing cells when exposed to cystine expectedly showed a large increase in cystine levels (Fig. 2B). This amount was maximal in 1 hour, but by 5 hours the levels of cystine reduced to basal levels. This suggests that although initially cystine accumulation occurs, cystine is removed and converted to other compounds. These same cells also showed a significant increase in cysteine amounts (Fig. 2B) suggesting that much of the cystine that was pumped inside was being converted to cysteine and potentially utilized.

### Estimation of absolute concentrations of cystine in minimal media grown yeast cells reveal that intracellular cystine is in the 50-100 nanomolar range

In minimal media (SD) grown cells the levels of cysteine have been estimated to be in the range of 100-400 nM 21. However, cystine levels have never been estimated. Since a ~200-fold increase in cystine was seen in some of the experiments we thought it was important to estimate intracellular cystine concentrations. We observed that the normal levels of cystine seen in a yeast cell grown in minimal media conditions was in the range of 50-100 nM, almost comparable to cysteine concentrations, and this increased up to ~10 µM concentration due to cysteine overload.

### Relative amounts of homocysteine, cystathionine, SAM and SAH show a delayed increase compared to cysteine

**Figure 3:**
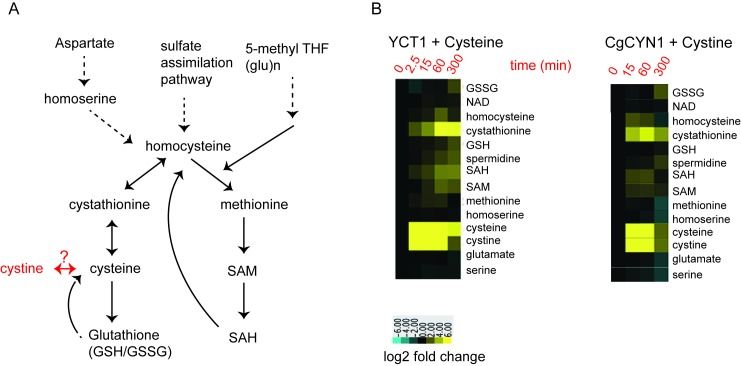
FIGURE 3. **(A)** A scheme of the sulfur amino acid metabolic pathway in yeast. **(B) **Heat maps illustrating a hierarchical clustering of changes in the indicated metabolites from cells expressing (i) TEF-*YCT1* treated with 500 µM cysteine and measured at 0, 2.5, 15, 60 and 300 min and (ii) TEF-*CgCYN1* treated with 500 µM cystine and measured at 0, 15, 60 and 300 min.

The sulfur assimilation and amino acid biosynthesis pathway is a highly interconnected pathway, with homocysteine as a key intermediate metabolite (Fig. 3A). We therefore measured other key metabolites in the sulfur amino acid pathway, starting with homocysteine, using a targeted, quantitative LC-MS/MS based approach, in cells overexpressing *YCT1 *and treated with a high dose (500 µM) of cysteine. We also used the same approach to measure these metabolites in cells overexpressing *CgCYN1* and treated with a high dose of cystine. Expectedly, there are substantial changes in the amounts of these metabolites, yet these metabolites show both distinct temporal profiles, as well as relative changes in their amounts (Fig. 3B). Importantly, the metabolites in the sulfur amino acid pathway increased in abundance over time in the order expected from their positions in the pathway (Figs. 3A and 3B), and the relative changes in amounts were entirely different for the different metabolites, ranging from several hundredfold for cysteine to much smaller changes for several other metabolites.

**Figure 4:**
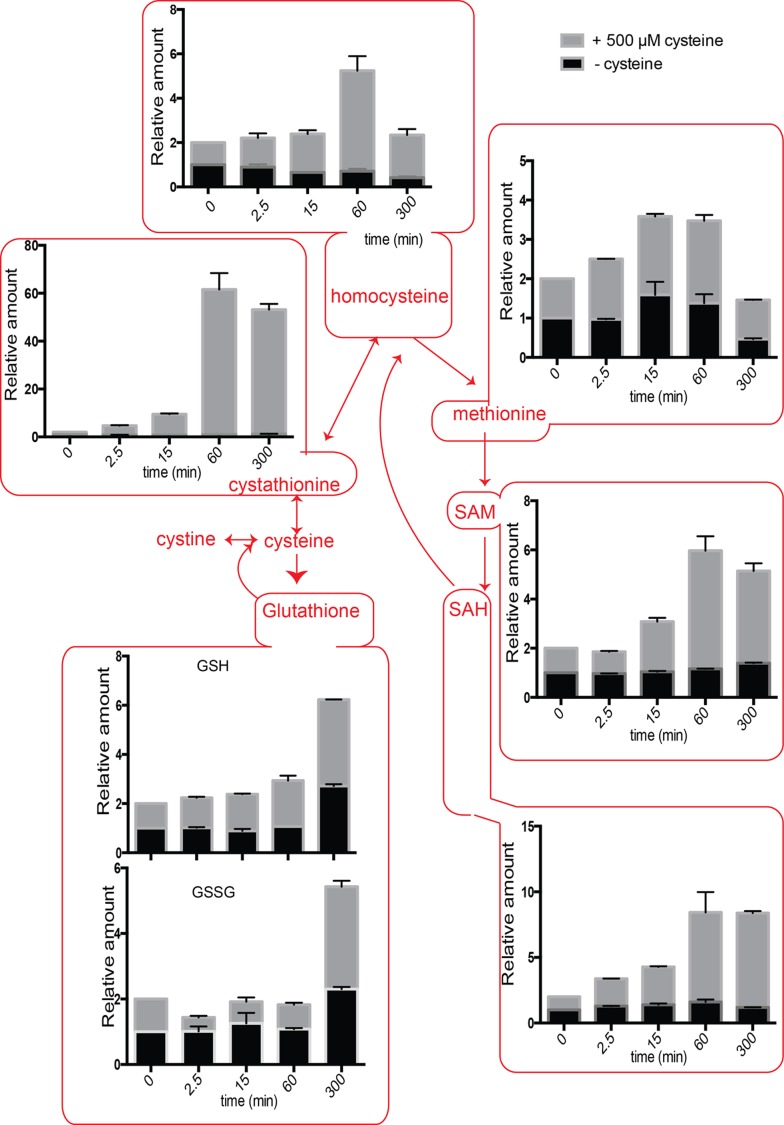
FIGURE 4. Positioned in the context of the sulfur amino acid pathway: Relative intracellular amounts of sulfur amino acid metabolites as a function of time in cells overexpressing the cysteine transporter *YCT1* and challenged with 500 µM cysteine.

The intracellular amounts of homocysteine increased after cysteine treatment to ~5-fold above normal, after ~1 hour. Eventually, this decreases to ~2-fold above normal after 5 hours (Fig. 4). When we examined cystathionine under these conditions, we observed that cysteine exposure led to a 60-fold increase in cystathionine concentrations (Fig. 4). Furthermore, even after 5 hours, the levels of cystathionine remained significantly higher than the control cells.

SAM (S-Adenosyl L-methionine) levels also increased up to ~5-fold and remained high till ~5 hours (Fig. 4). Similarly, SAH (S-Adenosyl L-homocysteine) amounts increased up to 7-fold with maximum values achieved at ~5 hours (Fig. 4). Considering that the normal levels of SAM are in the 10-30 µM, and SAH are in the range of 100-400 µM in *S. cerevisiae *21, these fold increases are quite substantial. In contrast, when we examined the relative levels of methionine in the cells accumulating cysteine, we observed that it led to only a ~2-fold change in methionine levels (Fig. 4), that returned to normal levels in about 5 hours. Methionine levels are normally in the range of 30-60 µM, and thus not much of the excess thiol was trapped in methionine. Indeed, there are other studies as well where sulfur amino acid metabolism change also indicates that cells maintain methionine concentrations within a fairly stable steady state range 21,23.

**Figure 5:**
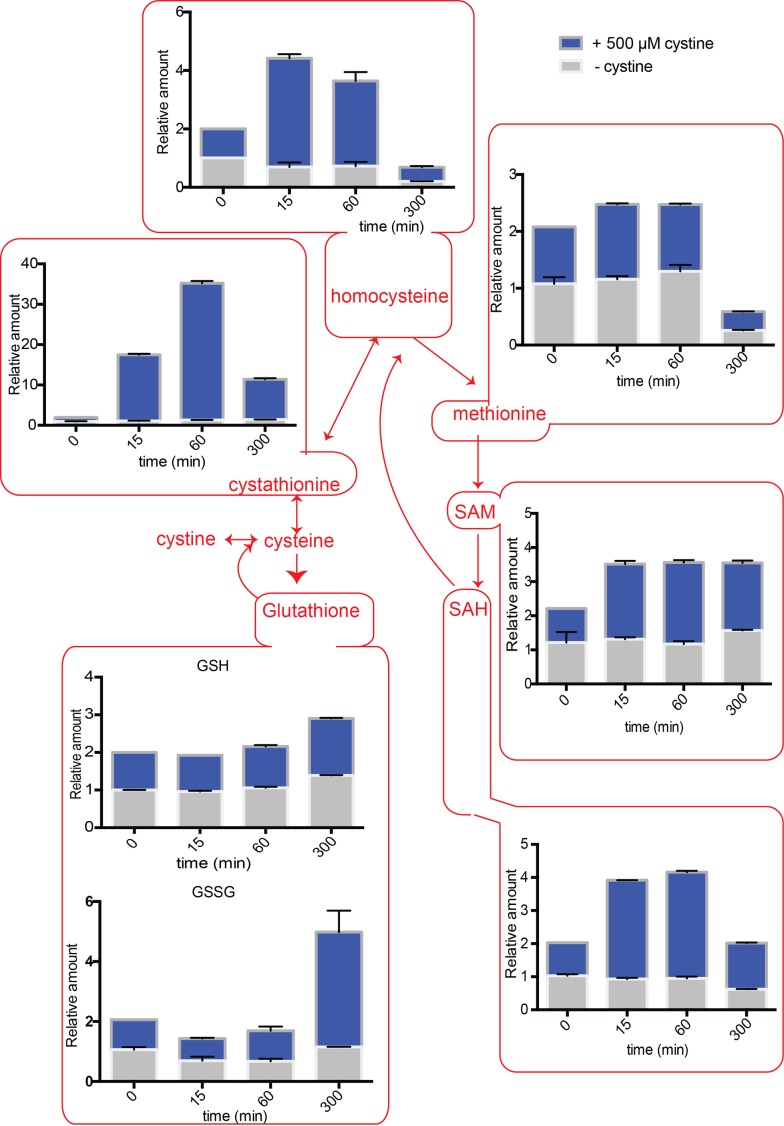
FIGURE 5. Positioned in the context of the sulfur amino acid pathway: Relative intracellular amounts of sulfur amino acid metabolites as a function of time in cells overexpressing the cystine transporter *CgCYN1* and challenged with 500 µM cystine.

Similar trends were observed when cystine was added to the cells expressing the *CgCYN1* transporter (Fig. 5), indicating an overall conserved metabolic output to acute addition of cysteine as well as cystine. However, the magnitude of increase in most metabolites was smaller. Interestingly, these data suggest how different metabolites in the sulfur amino acid pathways are maintained during basal homeostasis at entirely distinct concentration ranges, also reflecting why the relative change in amounts observed for each metabolite is likely to be entirely distinct, ranging from the ~100-fold changes for cysteine (normally maintained at very low absolute amounts) to single-fold changes for other metabolites present at much higher concentrations normally.

### Relative amounts of the naturally abundant GSH increase gradually to 3-fold higher than normal

We also observed a delayed increase in metabolites that could be considered long-term storehouses for sulfur containing amino acids. The total amounts of both GSH (glutathione) and GSSG (oxidized glutathione) did not change immediately after cysteine or cystine addition, but with time, in both the cysteine and cystine treated cells, the GSH and GSSG levels increased (Figs. 4 and 5). Although the fold increase was only about 3-fold, considering that GSH is found at very high concentrations between 1-10 mM in the cell, these changes are substantial.

Collectively, it is notable that different metabolites in the sulfur amino acid pathway show entirely different changes in abundance, both temporally as well as in the magnitude of change in relative abundance. These data reveal a sequential conversion and utilization of metabolites, as well as specific metabolites within the pathway that are maintained at more or less constant levels, upon encountering high cysteine (or cystine) levels in cells.

### Genes up-regulated with TEF-YCT1 bearing cells exposed to cysteine

Our data revealed immediate, acute metabolic responses to cysteine overload, followed by a steady redistribution of the metabolites in the sulfur amino acid pathway. We therefore set out to understand global transcriptional changes in yeast to cysteine and cystine overload, to explain how cells eventually adapted to increased amounts of cysteine-derived metabolites. Using a genome-wide expression profiling approach, we identified genes that were differentially regulated in cells exposed to excess cysteine. For these experiments, *S. cerevisiae *cells bearing a deletion of *YCT1* and constitutively over-expressing the cysteine transporter (TEF-YCT1) were grown in minimal medium and treated with 0.5 mM cysteine for 5 hours, and subsequently processed as described in Materials and Methods.

**Figure 6:**
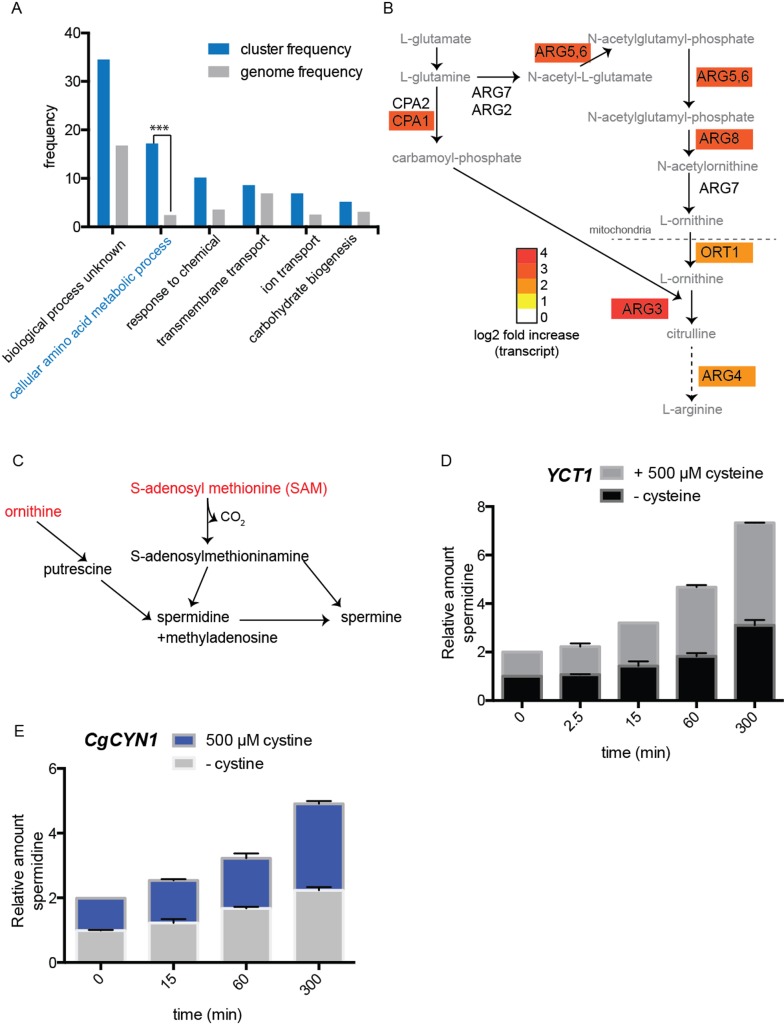
FIGURE 6. Gene expression changes due to cysteine or cystine addition to cells. **(A)** Gene ontology based analysis (by process), illustrating induced genes that are dramatically overrepresented (by process) upon cysteine addition. **(B)** A mapping of highly induced genes onto the ornithine/arginine biosynthesis pathway. The heat-map shows (log2) fold change in transcripts induced. **(C)** A schematic of the metabolic pathways used to make polyamines, spermidine and spermine, emphasizing the use of SAM (S-Adenosyl L-methionine) and ornithine in this process. **(D)** and **(E)** relative changes in spermidine amounts over time, upon the addition of cysteine (D) or cystine (E) to cells expressing *YCT1* or *CgCYN1* respectively.

Interestingly, only a small subset of ~58 genes in a few functional clusters, was globally up or down regulated, revealing that a limited, highly specific response was sufficient for cells to adapt to cysteine overload (Table S2). To validate the microarray data a few of the upregulated genes in the TEF-YCT1 treated cells were also analyzed by qPCR (Figure S1). Strikingly, a gene ontology mapping of these genes to biological process revealed a very strong overrepresentation in cellular amino acid metabolic processes (Fig. 6A). Particularly among this cluster, genes from the ornithine/arginine biosynthetic pathway were highly up-regulated (Fig. 6B). This included the ornithine carbamoyl transferase, acetyl ornithine transferase, acetyl glutamate kinase and N-acetyl-γ-glutamyl-phosphate reductase and arginine succinate lyase (*ARG3, ARG8, ARG5,6, *and *ARG4*) (Fig. 6B). Additionally, other genes related to arginine metabolism including the vacuolar cationic amino acid transporters that principally transport arginine, and the mitochondrial ornithine transporter that exports ornithine from the mitochondria to the cytoplasm (*RTC2* and *ORT1*) were also induced (Fig. 6B), all suggesting that the adaptation response to acute cysteine overload eventually induced the ornithine/arginine biosynthetic pathway, which is typically considered an anabolic pathway.

This was surprising, but suggested a possibility that would metabolically make sense. A major consumer of the high amounts of SAM made during the redistribution of cysteine to other metabolites in the sulfur amino acid pathway (Figs. 3, 4, and 5) could be the polyamine pathway (Fig. 6C). Spermidine and spermine are two abundant, major metabolites that consume substantial amounts of SAM (Fig. 6C). Importantly, this also requires ornithine (from the ornithine/arginine pathway, Fig. 6B). We therefore tested this hypothesis by directly measuring spermidine produced over time, post cysteine and cystine treatment. Spermidine showed a substantial increase in concentrations in the cysteine treated cells (Fig. 6D). Approximately 4-fold increase could be seen at the end of 5 hours. Considering the high levels of spermidine in the cell (2mM) 24, this signifies a very large increase in the absolute amounts of polyamines. Thus, it appears that one major destination (in addition to GSH) of the high amounts of cysteine are the polyamines, which would also require a simultaneous increase in the synthesis of ornithine as well as arginine, and together these might serve as anabolic molecules for subsequent growth.

Additionally, genes involved in iron and/or siderophore acquisition such as *FMP23*, *TIS11*, *FIT2*, *SIT1* and *ARN1* were found to be upregulated (Table S2). Relatedly, the enzyme trans-aconitate methyltransferase (*TMT1*) of the leucine biosynthetic pathway was also significantly upregulated, and this is known to be highly induced during iron limitation 25. Apart from these, *ZRT1* (high-affinity zinc transporter of plasma membrane) gene was up regulated that is known to be involved in maintaining zinc homeostasis in the cell (Table S2). Finally, two genes of the sulfur metabolic pathway genes, *STR2* (cystathionine gamma-synthase) and *SSU1* (sulfite efflux pump), were found to be up regulated (Table S2). One possible explanation could be some non-oxidative degradation similar to what has been observed in mammalian cells by cystathionine beta synthase and thus the sulfite efflux pump might also be required under these conditions. Indeed, the sulfite efflux pump has been shown to be important for cysteine tolerance in *Candida albicans *18. In addition several genes with unknown function such as YJL160c and *SRL4 *were upregulated. These are most likely to be associated with arginine biosynthesis, iron (or iron-sulfur) metabolism, or sulfur metabolism.

### Genes upregulated with TEF-CgCYN1 bearing cells exposed to cysteine

**Figure 7:**
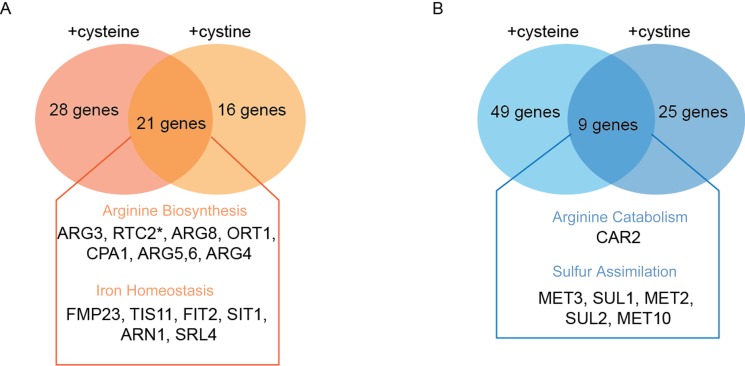
FIGURE 7. **(A)** Venn diagram showing a list of genes involved in specific pathways that are upregulated in *S. cerevisiae* cells expressing TEF-*YCT1* treated with cysteine (500µM), TEF-*CgCYN1* treated with cystine (500µM), where the genes commonly induced in both [p<0.05, fold change ≥ 2.0 (cysteine) and fold change ≥ 1.40 (cystine)] are emphasized. **(B)** Venn diagram showing a list of genes involved in specific pathways that are down regulated in *S. cerevisiae* cells expressing TEF-*YCT1* treated with cysteine (500µM), TEF-*CgCYN1* treated with cystine (500µM) and set of genes common to both [p<0.05, fold change ≥ 1.5(cysteine) and fold change ≥ 0.8 (cystine)].

In the case of the cystine overload experiments, we again observed an upregulation of the arginine biosynthetic pathway (Table S3, Fig. 7A). This included ornithine carbamoyl transferase (*ARG3*), acetyl glutamate kinase and N-acetyl-gamma-glutamyl phosphate reductase (*ARG5,6*), vacuolar cationic amino acid transporter (*RTC2*), acetyl-ornithine amino transferase (*ARG8*), small subunit of carbamoyl phosphate synthase (*CPA1*), mitochondrial ornithine transporter (*ORT1*), argininosuccinate lyase (*ARG4*) (Table S3). Similarly, we estimated changes in polyamine (spermidine) amounts after cystine treatment, and observed a substantial increase in spermidine post cystine treatment (Fig. 6E). These data matched the earlier data observed with cysteine treatment.

Similarly, genes involved in iron and/or siderophore acquisition and leucine biosynthesis were up-regulated as seen under cysteine treated cells. Mitochondrial succinate-fumarate transporter (*SFC1-*4.7-fold) was also found to be up regulated. Finally, two genes of the sulfur metabolism, cystathionine beta-lyase (*STR3*) and the sulfite efflux pump *SSU1*, and the zinc transporter *ZRT1 *were upregulated. The genes up-regulated due to cystine overload strikingly overlapped with the genes up-regulated due to the overload of cysteine (Fig. 7A), however their fold induction was lower and hence a lower cut off has been used. This correlates well with the observed metabolite data, and the relative changes in abundance of these metabolites, all suggesting a conserved response to cysteine and cystine, with a relatively modest response for cystine.

Collectively, for both cysteine and cystine overload, there is a striking overlap of genes that are induced (Fig. 7A). Particularly, the induced genes are more related to amino acid metabolism that leads towards anabolism, particularly the ornithine/arginine biosynthesis pathway (Fig. 7A), leading to a final increase in polyamines that we have observed. Other changes include reducing sulfite assimilation, as well as dealing with iron and zinc limitations (all known to occur under conditions of cysteine toxicity), and not directly related to primary sulfur amino acid metabolism.

### Genes down-regulated under conditions of cysteine or cystine overload in the cells

The down-regulated genes for the cysteine overload experiments (*YCT1*) (Table S4, Fig. 7B) included the genes from inorganic sulfur assimilation pathway such as the high affinity sulfate permease (*SUL1*), homoserine-O-acetyltransferase (*MET2*), ATP sulfurylase (*MET3*), sulfite reductase (*MET5*), adenylylsulfate kinase (*MET14*), and S-methyl methionine homocysteine methyltransferase (*MHT1*). All of these can be explained by a lack of the need to reduce and assimilate sulfur, which is no longer needed since cysteine provides a ready form of usable sulfur to cells. In case of the down-regulated gene list with cystine overload (*CgCYN1*) using the log2 cut-off of two, we did not observe any genes. However, on lowering the cut-off, some genes were indicated (Table S5). This list again included genes primarily from inorganic sulfur assimilation pathway and genes of the inorganic sulfur metabolic pathways such as *SUL1* (2.0-fold), *MET2* (2.1-fold), *MET3* (2.1-fold), *MET6* (1.8-fold), *MET10* (2.0-fold), as well as the glutathione transporter (*OPT1*/*HGT1*: 2.2-fold). Collectively, for both cysteine and cystine overload, there is a striking overlap of genes that are down regulated (Fig. 7B). All these data appear to be logical, since with an overload of cysteine, cells will no longer have to rely on challenging reductive biosynthesis of homocysteine from free sulfite.

### Supplementation of arginine, zinc or iron does not reverse cysteine toxicity

Our metabolite analysis suggests that cells deal with cysteine toxicity by converting cysteine to less reactive metabolites, which lead to multiple anabolic pathways. This would require cells to be able to assimilate these metabolites for anabolism. Importantly, our gene expression analysis revealed a strong upregulation of ornithine/arginine metabolism ~5 hours after the addition of cysteine, when these other metabolites derived from cysteine metabolism were most abundant. This suggested that the most likely role of the ornithine/arginine metabolic pathway would be to synthesize and assimilate arginine and arginine derived metabolites, particularly polyamines, which require SAM metabolism. However, to eliminate the remote possibility that arginine limitation might determine cysteine toxicity responses, growth assays were carried out with strains over-expressing cysteine transporter under the TEF promoter in media supplemented with a range of arginine concentrations (0.01 - 2 mM). The addition of the amino acid expectedly enhanced the growth of the strains, but had no effect on the toxicity of cysteine that resulted from cysteine overload (not shown). The strains were similarly tested for reversal of cysteine toxicity by either zinc or iron since these pathways were also induced during cysteine or cystine overload. We added these metal ions in concentrations ranging from 0.01 to 0.5 mM. However, none of these metals or growth conditions could rescue the toxicity to any discernable level (data not shown).

Collectively, our data permit an interpretation suggesting that the excess cysteine, which is immediately channeled to a number of less reactive thiols, and subsequently to metabolites such as SAM and GSH, can be effectively finally utilized for anabolic processes typically derived from arginine metabolism. We explain these possibilities in the discussion section.

### Investigation of deletion mutants in different pathways reveals no single enzyme is critical for survival under cysteine or cystine overload

Our data strongly suggests that the simultaneous occurrence of multiple metabolic processes allow cells to overcome cysteine overload. However, we wanted to determine if any one of the overexpressed gene clusters were particularly important for cells to handle cysteine toxicity. We utilized multiple strains from the Euroscarf collection (listed in Table S1) with deletions in the different pathways that were upregulated in response to excess cysteine or cystine. Particularly, these included the trans-sulfuration pathway (for cysteine to homocysteine conversion), and the arginine and the polyamine biosynthetic pathways. We also tested gene deletions of iron homeostasis and/or siderophore receptor synthesis that were upregulated.

Of particular interest were the single deletion mutants of the pathway from cysteine to homocysteine that included, *STR2 *(cystathionine gamma synthase) and *STR3 *(Cystathionine beta-lyase). However, none of these single mutants showed any enhanced toxicity as seen by plate assays. The *SSU1* sulfite efflux pump also did not show any enhanced toxicity suggesting that sulfite efflux was not the principal pathway being employed. We also evaluated the enzymes in polyamine biosynthesis that included *SPE1*, *SPE2*, *SPE3* and *SPE4*. No enhanced cysteine toxicity was observed. Finally, we tested mutants of iron/or siderophore acquisition that were upregulated with cysteine or cystine overload as well as several genes of unknown function. These genes included *FIT2*, *FMP23*, *SIT1*, *AFT1* and *AFT2, TMT1, *and the ORFs YGL117w and YEL057c. None of these mutants showed any enhanced toxicity in the cysteine toxicity experiments (data not shown). Collectively, these data strongly support our conclusion that no single gene or pathway is responsible for cells to handle cysteine overload, and multiple pathways allow the final assimilation of metabolites derived from cysteine.

## DISCUSSION 

In this manuscript, we first established a system of evaluating cysteine overload in yeast arising from high intracellular levels of cysteine without the need for addition of very high extracellular levels of the amino acid. This thereby enabled precise and systematic manipulations and thus is likely to better reflect the consequences of high intracellular cysteine accumulation. To sharpen our analysis, we additionally used the heterologously expressed *C. glabrata *cystine specific transporter (*CgCYN1*) in *S. cerevisiae, *which clarifies the metabolic consequences of the effects of cysteine influx. The effectiveness of the experimental setup was immediately apparent from the toxicity towards cysteine observed at lower media concentrations than previously reported. This was further validated when we observed that although relatively lower concentrations of these amino acids were added in the medium (10-fold lower than previous studies 17, we achieved high intracellular concentrations (over 600-fold higher than the steady-state, normal concentrations). Interestingly, the higher concentrations of cysteine also led to a large increase in intracellular cystine amounts within the cell, and vice versa. This provides a satisfying validation for the excellent correlation between the microarray experiment under both conditions, as well as the metabolite analysis carried out using similar conditions.

The observation that cysteine rapidly converts into cystine suggests that the reactive thiol of cysteine is quickly trapped in a less-reactive form. Cysteine was also subsequently distributed to other less-reactive thiols, such as cystathionine where a 60-fold increase in levels was seen. Over time, this was distributed among other thiols that included SAH, SAM, and also as GSH, all of which are present at concentrations several orders of magnitude greater than cysteine at normal steady state 21. Thus, it appears that cells convert excess cysteine to cystine and other inert thiols as a mechanism to detoxify, and possibly store excess cysteine, an otherwise valuable nutrient to the cells. This happens through systematic, temporally separated changes in the amounts of different thiol metabolites, with the reactive thiol of cysteine sequestered away by ‘thiol trapping’. It is possible that additional methods of detoxification that include sequestration into the vacuole of the oxidized cystine, might also exist in a manner similar to the sequestration of oxidized glutathione, GSSG, that plays an important role in glutathione redox homeostasis 26. However, this is more difficult to test experimentally, due to challenges in isolating metabolites from cellular compartments.

We suggest that such ‘thiol trapping’ to sequester and utilize cysteine is general to all organisms, and not just to organisms like *S. cerevisiae*, lacking the oxidative degradative pathway of cysteine. Considering that sulfur assimilation is an energy consuming process, it seems likely that organisms would preferentially store and recover such forms of reduced sulfur, rather than degrade and dispose them from the cell. It is reasonable to therefore suggest that this might be a primordial strategy adopted by living cells, with degradation and consumption coming as a subsequent step. Consistent with this hypothesis, it is interesting to note that in the mouse cysteine dioxygenase knockouts, the GSH levels increased approximately two-fold 27,28. Other thiol intermediates such as Cys-GSH mixed disulfides which may also form were, however, not examined. Nevertheless, the elevated GSH levels alone suggest that ‘thiol trapping’ might be an effective mechanism in metazoans as well, although not explicitly recognized as such, till now.

The metabolite estimations were carried out at early, intermediate and late time points, providing a temporal sequence of early and later metabolic events. However, the gene expression arrays were performed at the later time point (5 hours), to provide an understanding of the eventual homeostatic adaptations for tackling high levels of cysteine. These microarray results revealed that when high levels of cysteine and cystine accumulate, only one major pathway seems to be primarily induced - the ornithine/arginine biosynthesis pathway. However, it must be pointed out that this is based on the 5 hour time point data. At earlier time points, it is possible that other pathways might also be induced, but this was not examined, since this was not the purpose of this study. The increase in the expression of the mitochondrial ornithine transporter *ORT1* and the vacuolar cationic amino acid transporter *RTC2 *can also be explained by this conversion to arginine. Interestingly, *RTC2, *which has been shown to be upregulated by lysine limitation, preferentially transports arginine (as compared to lysine) 29. The results from this study indicate that *RTC2* might also be under regulation by arginine limitation as well. Accounting for the upregulation of the siderophore receptors, many of these siderophores have in their core metabolites derived from ornithine (N-hydroxyl ornithine), which is itself derived from arginine metabolism 30,31. Although *S. cerevisiae *lacks the siderophore biosynthetic pathway 32, the up-regulated siderophore receptors could bind and take up these siderophores present in the extracellular medium and thus further help fulfill the cellular arginine/ornithine requirement.

Importantly, a simple metabolic process can explain why ornithine/arginine genes might be upregulated over time. SAM is intimately linked to arginine and polyamine biosynthesis 33,34. One explanation for the increased requirement of arginine/ornithine is that some excess cysteine can be channeled into the polyamine biosynthesis pathway to form spermine and/or spermidine with ornithine/arginine as a precursor for biosynthesis. The ornithine/arginine pathway is linked to SAM through 5’-methylthioadenosine (MTA) formation, a byproduct of polyamine biosynthesis in the methionine salvage pathway 35. Importantly, the synthesis of spermidine is entirely SAM dependent, requiring SAM and decarboxylated SAM to convert putrescine (derived from ornithine/arginine) to spermidine 33,34. This simple feed-forward mechanism, converting reactive cysteine to SAM (observed in our data), and subsequently using this towards amino acid biosynthesis, arginine biosynthesis and consumption, polyamine biosynthesis and finally nucleotide biosynthesis will allow cells to restore growth after a time-period of reduced growth due to high cysteine concentrations, and thereby utilize a valuable, scarce resource. Polyamines themselves are both highly abundant and critical metabolites for growth, important for a variety of reasons 36,37,38 and so would be an effective final outcome when cysteine is converted into more usable forms of protected thiols. Indeed, our data showed a substantial increase in intracellular polyamine concentrations (as measured directly by spermidine amounts) in ~5 hours after cells encounter large concentrations of cysteine.

In the case of cysteine overload cells we observed, in addition to *SSU1*, the *STR2* gene being upregulated. *STR2* encodes for the gene cystathionine gamma synthase. Cysteine metabolism proceeds through *STR2*, the first enzyme that acts on cysteine to take it to cystathionine, and then subsequently to homocysteine. Surprisingly, though, the *str2*Δ did not lead to any significant enhancement of sensitivity to cysteine toxicity. *STR2* deletions are otherwise unable to utilize cysteine as a sulfur source. These data suggest that cysteine is not as toxic in yeast as has been presumed. Furthermore, this supports the conclusion that cysteine has multiple routes to detoxification. Particularly, our data suggest that conversion of cysteine to cystine, along with conversion to GSH may be an effective mechanism to remove excess cysteine.

An earlier report about homocysteine and cysteine toxicity showed that cells reduce toxicity by means of upregulation of genes involved in primary metabolism, such as glycolysis, folate metabolism, and serine biosynthesis. The study also suggested that the cysteine and homocysteine toxicity is due to elevated ER stress related genes 17. In case of *E. coli, *growth inhibitory effects were apparently caused by inhibition of threonine deaminase by cysteine leading to isoleucine starvation and can be reversed by addition of isoleucine, leucine and valine 39. In contrast, in neurons it is reported that the cysteine toxicity is a result of hydroxyl radicals generated during the auto-oxidation of cysteine generating cystine and is catalyzed by copper, iron and other transition metals and can be reversed by catalase and pyruvate 20. In yeasts, in contrast, an oxidative stress was not observed, although an ER stress response was shown to occur, but this was done at much higher concentrations of externally added cysteine that could interfere with transport of other amino acids and thus could be a result of secondary consequences 17. However, none of these mechanisms appeared important based on the studies described here. Instead, some of these can be explained by textbook metabolic feed-forward loops 40, which would be consistent with the actual metabolite data observed in this study. In particular, increased folate metabolism can be expected if there is an increase in SAM availability, and increased folate metabolism is central for nucleotide biosynthesis through SAM dependent one-carbon metabolism, which also requires ribose and deoxyribose sugars from the pentose phosphate pathway branching from glycolysis 40.

Finally, whether the mechanism by which the oxidation of cysteine to cystine occurs is purely non-enzymatic, or might involve the possibility of an active enzymatic regulation remains unclear. Enzymatic conversion has been shown to occur through copper or copper-dependent proteins 41. Further, determination of *in vitro *cystine reduction activity by using purified thioredoxins, glutaredoxins, or screens leading to as yet unknown proteins might reveal proteins specifically involved in cystine reduction.

## MATERIALS AND METHODS

### Chemicals and Reagents

All chemicals used were obtained from commercial sources and were of analytical grade. Media components, fine chemicals and reagents were purchased from Sigma Aldrich (St. Louis, USA), HiMedia (Mumbai, India), Merck Millipore India Ltd (Mumbai, India), USB Corporation (Ohio, USA) or Difco, USA. Cysteine and cystine were also purchased from Sigma Aldrich (St. Louis, USA). RNA*later*® solution was procured from Thermo Fisher Scientific (Waltham, MA USA). Cysteine stock solutions that were prepared fresh were made by dissolving the required amount of cysteine in 1 ml of de-ionized water, which was then filter-sterilized using 0.2 μm filter membrane. Cystine stock solutions were prepared by dissolving the required amount of cystine in 1 ml of deionized water along with 25 µL of concentrated HCl, filter-sterilized using 0.2 μm filter membrane.

### Strains and Plasmids

*S. cerevisiae *strain MATα *ura3*Δ*0 leu2*Δ*0 his3*Δ*1 lys2*Δ*0 yct1*Δ*::kanMX4* (ABC1738) was used as the parent host for all the transformations, and was obtained from Euroscarf (Y11543). This strain and all the other *S. cerevisiae* strains used in this study are listed in Table S1. The yeast strains were maintained on yeast extract (1%), peptone (2%) and dextrose (2%) (YPD) medium and grown at 28-30(C. The yeast transformants were selected and maintained on synthetic defined minimal medium containing yeast nitrogen base (0.17%), ammonium sulfate (0.5%) and dextrose (2%) supplemented with histidine, leucine, lysine, and uracil at 80 mg/l as per requirement.

TEF-YCT1 (ABE2196) contained a C-terminal HA-tagged YCT1 downstream of the TEF-promoter in p416TEF vector. TEF-CgCYN1 (ABE2847) contained a C-terminal HA-tagged CgCYN1 downstream of the TEF promoter in the centromeric vector p416TEF.

### Cell growth and spotting experiments

For the growth assays, the *S. cerevisiae* strain (ABC1738) constitutively over-expressing TEF-YCT1 or TEF-CgCYN1 were grown overnight in SD minimal medium at 30(C at 220 rpm shaking, and inoculated in fresh medium to an OD_600_ of 0.1 and grown until the OD_600_ reaches 0.5 - 0.8. The cell suspensions (4 X 10^7^ cells) were serially diluted to 1:10, 1:100 and 1:1000. 10 µl of these cell re-suspensions were spotted on minimal medium containing different concentrations of cysteine or cystine (0.01-1 mM). The plates were incubated at 30(C for approximately 2 days.

### Total metabolite extraction and preparation

For metabolite analysis, the *S. cerevisiae *(ABC1738) transformants which were constitutively over-expressing cysteine transporter (*YCT1*) or cystine transporter (*CgCYN1*) were exposed to 500 µM cysteine or cystine. Samples were collected at different time intervals (0, 2.5, 15, 60, 300 min) and all samples except the zero time point were done in duplicate. Samples were rapidly quenched in a quenching solution, following which metabolite extraction was done. Metabolite quenching and extraction was performed as described previously 23,42. Metabolite extracts were dried down in a speed-vacuum (3-4 hours), and stored at -80°C until analyzed by mass spectrometer.

### Targeted metabolite analysis

For detecting metabolites, a triple-quadrupole mass spectrometer (TSQ Vantage, Thermo Scientific) coupled to an HPLC was used, which allows one to detect each metabolite simultaneously using multiple reaction monitoring (MRM). Metabolites were separated chromatographically using a Synergi Fusion column (150 × 2.0 mm, 4 μm, Phenomenex), using a Shimadzu Prominence HPLC (high-performance liquid chromatography) autosampler coupled to the mass spectrometer. Extracted metabolites were measured using targeted LC-MS/MS methods, detailed method described previously 23,42. A library of common metabolites was constructed using standards, and metabolites were detected using a TSQ Vantage (Thermo Scientific) triple quadrupole-linear ion trap mass spectrometer for quantitative optimized detection of daughter ions upon collision-induced fragmentation of the parent ion. For each metabolite, parameters for quantitation of the two most abundant daughter ions (that is, two MRMs per metabolite) were included. To quantify metabolites, the area under each peak was quantitated by using the Xcaliber software, inspected for accuracy, and normalized against total ion count, after which relative amounts were quantified.

### Estimation of intracellular metabolite concentrations

Measurement of absolute cystine concentrations in the yeast cells was done using transformants expressing *YCT1* which yielded highest fold change (200-fold) of cystine in the cells upon cysteine overload. LC-MS/MS system Agilent 1200 infinity series connected to a TSQ vantage (Triple quadrapole) Mass spectrometer which uses Electrospray Ionization (ESI) was used for detection of intracellular cystine. The samples were separated by the LC-MS/MS system using a synergy RP-fusion column. An MRM method was created for the targeted metabolite. Buffers used for positive mode analysis were: Buffer A 99.9% H_2_O/0.1% formic acid, and Buffer B- 99.9% methanol/0.1% formic acid (T = 0 min, 0% B; T = 3 min, 5% B; T = 12 min, 80% B, T = 12.1 min, 90% B, T = 13 min, 0% B, T = 15 min, stop). Standards of known cystine concentrations were run under the same conditions and the area under the peak for each concentration was used to make a standard plot. The area under each peak was quantitated by using the Xcaliber software, inspected for accuracy, and the concentration of cystine extrapolated from the standard curve for a given volume of cells (≈10 ml or 9.38 OD). The intracellular concentration of cystine was estimated assuming the volume of a single yeast cell = 42 fl 43 and 1 OD = 2.0 X 10^7^ cells.

### Microarray analysis: growth of cells, RNA extraction and analysis

#### Growth of cells

The *S. cerevisiae* (ABC 1738) transformants constitutively over-expressing either cysteine transporter (TEF-YCT1) or cystine transporter (TEF-CgCYN1) were grown overnight in SD minimal medium, followed by secondary inoculation at 0.1 OD cells in same medium. After 3 hours of incubation at 30°C, we added either 500 µM cysteine or cystine, whereas respective control samples were grown without any further additions. After 5 hours of incubation of both test and respective control samples, cells were harvested, washed and re-suspended in RNA*later® *(10^8 ^cells). Four samples were in the cysteine analysis group (2 test samples and 2 control), and four samples were in the cystine treated group (2 test and 2 control).

#### RNA isolation

Total RNA was extracted using Qiagen’s RNeasy minikit (Cat#74104) following the manufacturer’s protocol. Total RNA integrity was assessed using RNA 6000 Nano Lab kit on the 2100 Bioanalyzer (Agilent, Palo Alto, CA) according to the manufacturer’s protocol. Total RNA concentration was measured on the NanoDrop® ND-1000 UV-Vis Spectrophotometer (Nanodrop technologies, Rockland, USA). Total RNA with OD_260_/OD_280_>1.8 and OD_260_/OD_230_ ≥ 1.1 was used for microarray analysis.

#### cRNA synthesis, labeling and hybridization

The purified RNA samples were further processed for One-Color Microarray-Based Gene Expression Analysis. As per the manufacturer’s protocol Agilent’s Quick-Amp labeling Kit (Cat#5190-0442) was used for the cRNA synthesis and Cyanine-3 CTP labeling. Qiagen’s RNeasy mini spin columns were used for purifying amplified cRNA samples. The quantity and specific activity of cRNA was determined by using NanoDrop ND-1000 Spectrophotometer version 3.2.1. Samples with specific activity >8 were used for hybridization. Hybridization solution was prepared using the Agilent’s *in situ *Hybridzation kit (Cat#5190-0404). The resulting mixture was applied to the Yeast (V2) Gene Expression Microarray, 8x15K (Agilent Technologies AMADID: 16322), and hybridized at 65°C for 16 hours in the hybridization oven. After hybridization, slides were washed with Agilent Gene expression Wash Buffer-I for 1 min at room temperature followed by 1 min wash with Wash Buffer-II at 37°C. Slides were finally rinsed with acetonitrile for cleaning up and drying. These experiments were carried out at Genotypic Co., Bangalore, India.

#### Microarray scanning and data extraction

Hybridized arrays were scanned at 3μm resolution on an Agilent Microarray Scanner, Model G2565BA. The Images were manually verified and found to be devoid of uneven hybridization, streaks, blobs and other artifacts. Hybridization across the slides was good based on number of features that were positive and significantly above background. Data extraction from Images was done using Feature Extraction software of Agilent (V-11.5). Percentile shift Normalization was done using GeneSpring GX version 13. One-fold and above differentially regulated genes were filtered from the data and were considered for further analysis. With respect to control sets, fold change was calculated (logbase2) and genes showing fold change of ±0.6 were further considered for analysis. In order to identify the genes whose expression was affected in samples treated with cysteine, we used a cut off of log2 value of 2, which reflects the 4-fold change in expression of genes. We used lower cut off values for the samples treated with cystine, since the overall expression levels were lower. Only differentially regulated genes with a P≤0.05 were considered in the analysis. Microarray data has been submitted to the Gene Expression Omnibus (GEO) database (ACCID: GSE87794).

### RNA isolation and RT-PCR

RNA isolation and cDNA synthesis was done as described previously 44. The *S. cerevisiae* (ABC 1738) transformants constitutively over-expressing cysteine transporter (TEF-YCT1) were grown overnight in SD minimal medium, followed by secondary inoculation at 0.1 OD cells in same medium. After 3 hours of incubation at 30ᵒC, we added 500 µM cysteine to the test samples, whereas control samples were grown without any further additions. After 5 hours of incubation of both test and respective control samples, cells were harvested. Total RNA was isolated by hot acid phenol method using 15 ml phase lock gel heavy tubes (5 prime), followed by DNase I treatment (Cat#M6101, Promega) for 15 min at 30°C. Zymo-Spin II column (Zymo Research, Cat#C1008-250) was used for clean-up of RNA. cDNA synthesis from 3 µg total RNA was done using reverse transcriptase (RT) and random-hexamer primers (GoScript™ Reverse Transcription System (Cat#A5000) Promega) at 42°C for 16 hours. Real time quantitative PCR (RT-qPCR) was carried out on LightCycler® 480 II System (Roche molecular Diagnostics) by using SYBR green dye-based reagents (Maxima Sybr Green QPCR Master Mix (Cat#K0251) Thermo Fisher). List of primers used for the analysis are listed in Table S8.

## SUPPLEMENTAL MATERIAL

Click here for supplemental data file.

All supplemental data for this article are also available online at http://microbialcell.com/researcharticles/thiol-trapping-and-metabolic-redistribution-of-sulfur-metabolites-enable-cells-to-overcome-cysteine-overload/.
