# Supplementary material for: Thiol trapping and metabolic redistribution of sulfur metabolites enable cells to overcome cysteine overload
Source: Microb Cell. 2017 Mar 27;4(4):112–26. doi: 10.15698/mic2017.04.567 (PMC5376351; doi:10.15698/mic2017.04.567)
Supplement: Supplementary file 1 [file mic-04-112-s01.pdf]

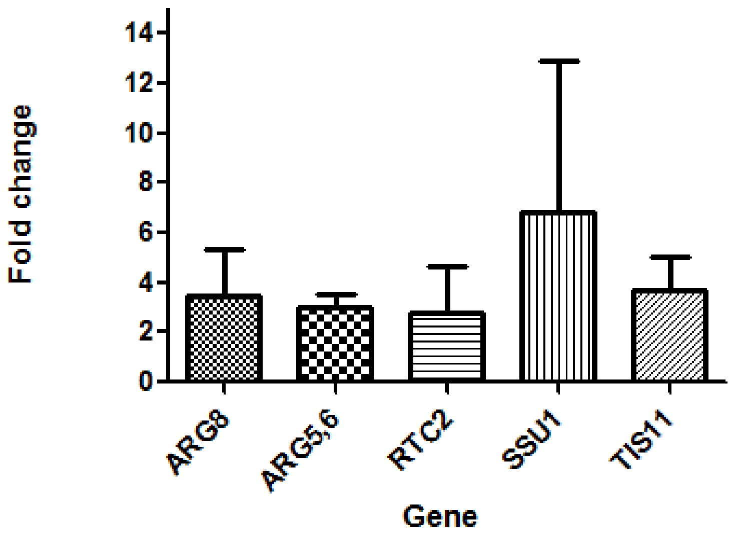

Fig.S1 Real-time PCR analysis of select genes from different metabolic pathways

S. cerevisiae transformants expressing TEF-YCT1 were either exposed to 500µM cysteine or no cysteine for 5 hours (Control samples). Total RNA was extracted followed by cDNA synthesis. RT-PCR analysis was done using SYBR green dye-based method. The experiment was carried out twice and the figure represents expression levels of the above mentioned genes in treated samples relative to the corresponding untreated control samples. The data above are means ± SD from technical replicates (n=3) of the biological duplicates. [ARG8, ARG5,6 and RTC2 belong to the arginine biosynthetic pathway, SSU1 is a part of sulphur metabolism and TIS11 is a gene involved in maintaining iron homeostasis in the cells.]

Table S1: List of strains used in this study

Strain	Genotype	Source
ABC 1738 (*yct1Δ)*	*MATα his3Δ1 leu2Δ0 lys2Δ0 ura3Δ0 YLL055W:: kanMX4*	Euroscarf (Y01543)
ABC3612(*arg3Δ*)	*MATa his3Δ1 leu2Δ0 ura3Δ0 YJL088w::kanMX4*	Euroscarf (Y11335)
ABC4812(*arg5,6Δ*)	*MATa his3Δ1 leu2Δ0 ura3Δ0 YER069w::kanMX4*	Euroscarf (Y10209)
ABC4811(*arg8Δ*)	*MATa his3Δ1 leu2Δ0 ura3Δ0 YOL140w::kanMX4*	Euroscarf (Y17711)
ABC4814(*spe1Δ*)	*MATa his3Δ1 leu2Δ0 ura3Δ0 YKL184w::kanMX4*	Euroscarf (Y15034)
ABC4815(*spe2Δ*)	*MATa his3Δ1 leu2Δ0 ura3Δ0 YOL052c::kanMX4*	Euroscarf (Y11743)
ABC4816(*spe3Δ*)	*MATa his3Δ1 leu2Δ0 ura3Δ0 YPR069c::kanMX4*	Euroscarf (Y15488)
ABC4817(*spe4Δ*)	*MATa his3Δ1 leu2Δ0 ura3Δ0 YLR146c::kanMX4*	Euroscarf (Y16945)
ABC3604(*ssu1Δ*)	*MATa his3Δ1 leu2Δ0 ura3Δ0 YPL092w::kanMX4*	Euroscarf (Y12160)
ABC1486(*str2Δ*)	*MATa his3Δ1 leu2Δ0 ura3Δ0 YJR130c::kanMX4*	Euroscarf (Y12608)
ABC4818(*str3Δ*)	*MATa his3Δ1 leu2Δ0 ura3Δ0 YGL184c::kanMX4*	Euroscarf (Y17556)
ABC3610(*aft1Δ*)	*MATa his3Δ1 leu2Δ0 ura3Δ0 YGL071w::kanMX4*	Euroscarf (Y14438)
ABC3611(*aft2Δ*)	*MATa his3Δ1 leu2Δ0 ura3Δ0 YPL202c::kanMX4*	Euroscarf (Y11090)
ABC4820(*tis11Δ*)	*MATa his3Δ1 leu2Δ0 ura3Δ0 YLR136c::kanMX4*	Euroscarf (Y14093)
ABC4821(*fit2Δ*)	*MATα his3Δ1 leu2Δ0 ura3Δ0 YOR382w::kanMX4*	Euroscarf (Y11679)
ABC4822(*sit1Δ*)	*MATa his3Δ1 leu2Δ0 ura3Δ0 YEL065w::kanMX4*	Euroscarf (Y10307)
ABC3609(*fmp23Δ*)	*MATa his3Δ1 leu2Δ0 ura3Δ0 YBR047w::kanMX4*	Euroscarf (Y13184)
ABC4824(*tmt1Δ*)	*MATa his3Δ1 leu2Δ0 ura3Δ0 YER175c::kanMX4*	Euroscarf (Y16171)
ABC1092(*zrt1Δ*)	*MATa his3Δ1 leu2Δ0 ura3Δ0 YGL255w::kanMX4*	Euroscarf (Y14622)
ABC4827(*srl4Δ*)	*MATa his3Δ1 leu2Δ0 ura3Δ0 YPL033c::kanMX4*	Euroscarf (Y12795)
ABC4742(*arg5,6Δ*)	*MATa his3Δ1 leu2Δ0 ura3Δ0 met15Δ0 YER069w::kanMX4*	Euroscarf (Y00209)

ABC4741(*arg8Δ*)	*MATa his3Δ1 leu2Δ0 ura3Δ0 met15Δ0 YOL140w::kanMX4*	Euroscarf (Y07711)
ABC4744(*spe1Δ*)	*MATa his3Δ1 leu2Δ0 ura3Δ0 met15Δ0 YKL184w::kanMX4*	Euroscarf (Y05034)
ABC4745(*spe2Δ*)	*MATa his3Δ1 leu2Δ0 ura3Δ0 met15Δ0 YOL052c::kanMX4*	Euroscarf (Y01743)
ABC4746(*spe3Δ*)	*MATa his3Δ1 leu2Δ0 ura3Δ0 met15Δ0 YPR069c::kanMX4*	Euroscarf (Y05488)
ABC4747(*spe4Δ*)	*MATa his3Δ1 leu2Δ0 ura3Δ0 met15Δ0 YLR146c::kanMX4*	Euroscarf (Y06945)
ABC4106(*str3Δ*)	*MATa his3Δ1 leu2Δ0 ura3Δ0 met15Δ0 YGL184c::kanMX4*	Euroscarf (Y07556)
ABC4752(*fit2Δ*)	*MATa his3Δ1 leu2Δ0 ura3Δ0 met15Δ0 YOR382w::kanMX4*	Euroscarf (Y01679)
ABC4751(*sit1Δ*)	*MATa his3Δ1 leu2Δ0 ura3Δ0 met15Δ0 YEL065w::kanMX4*	Euroscarf (Y00307)
ABC4754(*tmt1Δ*)	*MATa his3Δ1 leu2Δ0 ura3Δ0 met15Δ0 YER175c::kanMX4*	Euroscarf (Y06171)
ABC4761(*icy2Δ*)	*MATa his3Δ1 leu2Δ0 ura3Δ0 met15Δ0 YPL250c::kanMX4*	Euroscarf (Y01042)
ABC4756	*MATa his3Δ1 leu2Δ0 ura3Δ0 met15Δ0 YGL117w::kanMX4*	Euroscarf (Y04484)
ABC4757	*MATa his3Δ1 leu2Δ0 ura3Δ0 met15Δ0 YLR152c::kanMX4*	Euroscarf (Y04109)
ABC4758	*MATa his3Δ1 leu2Δ0 ura3Δ0 met15Δ0 YDL199c::kanMX4*	Euroscarf (Y03897)
ABC4759	*MATa his3Δ1 leu2Δ0 ura3Δ0 met15Δ0 YEL057c::kanMX4*	Euroscarf (Y00298)
ABC4760	*MATa his3Δ1 leu2Δ0 ura3Δ0 met15Δ0 YJL160c::kanMX4*	Euroscarf (Y07009)
ABC4762	*MATa his3Δ1 leu2Δ0 ura3Δ0 met15Δ0 YHR138c::kanMX4*	Euroscarf (Y01966)

Table S2: List of genes found to be up regulated in presence of cysteine

Gene Name	GmeanF	Fold change	Pathway	Description
ARG3	4.18	18.13	Arginine biosynthesis	Ornithine carbamoyltransferase
FMP23	4.17	18.00	Iron homeostasis	Putative protein of unknown function
TIS11	3.83	14.22	Iron homeostasis	mRNA-binding protein expressed during iron starvation
SRL4	3.44	10.85	Unknown	unknown function; regulation of dNTP

				production
YJL160C	3.37	10.34	Unknown	Putative protein of unknown function;
TMT1	3.24	9.45	Leucine biosynthesis	Trans-aconitate methyltransferase
ANB1	3.19	9.13	Translation	Translation elongation factor eIF-5A
RTC2	3.18	9.06	Arginine biosynthesis	YPQ3 vacuolar cationic amino acid transporter
ARG8	3.18	9.06	Arginine biosynthesis	Acetylornithine aminotransferase,
ZRT1	3.13	8.75	Zinc Homeostasis	High-affinity zinc transporter of the plasma membrane
PKP2	3.04	8.22	Pyruvate metabolism	Mitochondrial protein kinase
CPA1	3.04	8.22	Arginine biosynthesis	Small subunit of carbamoyl phosphate synthetase
SSU1	3.01	8.06	Sulfur metabolism	Plasma membrane sulfite pump
CRF1	3.00	8.00	Transcriptional regulation	Transcriptional corepressor;
FIT2	2.96	7.78	Iron homeostasis	Mannoprotein that is incorporated into the cell wall
ARG5,6	2.88	7.36	Arginine biosynthesis	Acetylglutamate kinase and N-acetyl-gamma-glutamyl-phosphate reductase
NCA3	2.76	6.77	ATP synthesis	Regulates mitochondrial expression of subunits 6 (Atp6p) and 8 (Atp8p) of the Fo-F1 ATP synthase
YEL057C	2.72	6.59	Telomere maintenance	Protein of unknown function
YLR152C	2.71	6.54	Unknown	Putative protein of unknown function
ICY2	2.67	6.36	Unknown	Putative protein of unknown function
YGL117W	2.63	6.19	Unknown	Putative protein of unknown function
ADH5	2.63	6.19	Amino acid degradation	Alcohol dehydrogenase isoenzyme
BSC5	2.58	5.98	Unknown	Putative protein of unknown function
GCY1	2.53	5.78	Glycerol metabolism	Putative NADP(+) coupled glycerol dehydrogenase,
SIT1	2.42	5.35	Iron homeostasis	Ferrioxamine B transporter
GLG1	2.40	5.28	Glycogen biosynthesis	Self-glucosylating initiator of glycogen synthesis

STR2	2.39	5.24	Sulfur metabolism	Cystathionine gamma-synthase,
ENB1	2.35	5.10	Iron homeostasis	Endosomal ferric enterobactin transporter
LYS20	2.34	5.06	Lysine biosynthesis	Homocitrate synthase isozyme
YDL199C	2.32	4.99	Unknown	Putative transporter, member of the sugar porter family
YSW1	2.31	4.96	Unknown	required for normal prospore membrane formation
YMR317W	2.28	4.86	Unknown	Putative protein of unknown function
AAD10	2.25	4.76	Amino acid metabolism	Putative aryl-alcohol dehydrogenase
ORT1	2.19	4.56	Arginine biosynthesis	Mitochondrial Ornithine transporter,
LYS1	2.19	4.56	Lysine biosynthesis	Saccharopine dehydrogenase
ARN1	2.19	4.56	Iron homeostasis	ARN family transporter for siderophore-iron chelates
NCE103	2.18	4.53	Non-classical protein export pathway	Carbonic anhydrase;
VMR1	2.17	4.50	Multidrug Resistance	Vacuolar membrane protein
YOR186W	2.17	4.50	Unknown	Putative protein of unknown function
FIG2	2.15	4.44	Cell wall maintenance	Cell wall adhesion
ECM13	2.14	4.41	Cell wall organization	Protein of unknown function
PEX21	2.10	4.29	Peroxisome biogenesis	Required for targeting of peroxisomal matrix proteins
YGP1	2.08	4.23	Unknown	Cell wall-related secretory glycoprotein;
YHI9	2.07	4.20	Unknown	Protein of unknown function
ARG4	2.07	4.20	Arginine biosynthesis	Argininosuccinate lyase
BOP2	2.07	4.20	Unknown	Protein of unknown function
YPL113C	2.05	4.14	Unknown	Glyoxylate reductase
BAP2	2.01	4.03	Leucine uptake	High-affinity leucine permease
RTS3	2.00	4.00	unknown	Put. component of protein phosphatase type 2A complex

Table S3: List of genes found to be up regulated in presence of cystine

Gene Name	GmeanF	Fold change	Pathway	Description
TIS11	3.57	11.88	Iron homeostasis	mRNA-binding protein expressed in iron starvation
FMP23	3.14	8.82	Iron homeostasis	Putative protein of unknown function
ARG3	2.85	7.21	Arginine biosynthesis	Ornithine carbamoyltransferase
FIT2	2.38	5.21	Iron homeostasis	Mannoprotein that is incorporated into the cell wall
TMT1	2.25	4.76	Leucine biosynthesis	Trans-aconitate methyltransferase
SFC1	2.22	4.66	Gluconeogenesis	Mitochondrial succinate-fumarate transporter,
RTC2	2.10	4.29	Arginine biosynthesis	YPQ3 vacuolar cationic amino acid transporter
ARG5,6	2.07	4.20	Arginine biosynthesis	Acetylglutamate kinase and N-acetyl-gamma-glutamyl-phosphate reductase
YGL117W	1.94	3.84	Unknown	Putative protein of unknown function
YLR152C	1.87	3.66	Unknown	Putative protein of unknown function
ARN1	1.86	3.63	Iron homeostasis	Arn1p specifically recognize siderophore-iron chelates
SRL4	1.80	3.48	Iron homeostasis	Unknown function; regulation of dNTP production
ARG8	1.77	3.41	Arginine biosynthesis	Acetylornithine aminotransferase,
ANB1	1.73	3.32	Translation	Translation elongation factor eIF-5A
TMA10	1.71	3.27	Unknown	Protein of unknown function,associates with ribosomes
ICY2	1.70	3.25	Unknown	Protein of unknown function
PKP2	1.68	3.20	Pyruvate metabolism	Mitochondrial protein kinase
CPA1	1.67	3.18	Arginine biosynthesis	Small subunit of carbamoyl phosphate synthetase
ORT1	1.64	3.12	Arginine biosynthesis	Exports ornithine from mitochondria
YLR194C	1.63	3.10	Cell wall protein	Structural constituent of the cell wall
ZRT1	1.62	3.07	Zinc Homeostasis	High-affinity zinc transporter of the plasma membrane,
STR3	1.62	3.07	Sulfur metabolism	Cystathionine beta-lyase, converts cystathionine into homocysteine
EST1	1.56	2.95	Telomere maintenance	TLC1 RNA-associated factor involved in telomere length regulation
SIT1	1.49	2.81	Iron homeostasis	Ferrioxamine B transporter,
SSU1	1.48	2.79	Sulphur metabolism	Plasma membrane sulfite efflux pump
YDR034W-B	1.46	2.75	Unknown	Protein of unknown function
SOL4	1.43	2.69	Pentose phosphate	6-phosphogluconolactonase with similarity to

			pathway	Sol3p
YHR138C	1.43	2.69	Unknown	Putative protein of unknown function
SNO4	1.42	2.68	Unknown	Possible chaperone and cysteine protease
GOT1	1.41	2.66	Secretory trafficking	Homodimeric protein packaged into COPII vesicles
NQM1	1.41	2.66	Unknown	Transaldolase of unknown function
ARG4	1.40	2.64	Arginine biosynthesis	Argininosuccinate lyase
YNR034W-A	1.38	2.60	Unknown	Putative protein of unknown function
YGL024W	1.35	2.55	Unknown	Dubious open reading frame
DDI2	1.35	2.55	Unknown	Protein of unknown function
HSP32	1.34	2.53	Unknown	Possible chaperone and cysteine protease
YGR109W-A	1.34	2.53	Transposable element	Retrotransposon TYA Gag gene

Table S4: List of genes found to be down regulated in presence of cysteine

Gene Name	GmeanF	Fold change	Pathway	Description
MET14	-4.59	24.08	Sulfur metabolism	Adenylylsulfate kinase, required for sulfate assimilation and involved in methionine metabolism
MET3	-3.81	14.03	Sulfur metabolism	ATP sulfurylase,
SUL1	-3.39	10.48	Sulfur metabolism	High affinity sulfate permease;
SPL2	-3.20	9.19	Unknown	Protein with similarity to cyclin-dependent kinase
MET5	-3.19	9.13	Sulfur metabolism	Sulfite reductase beta subunit
MET2	-3.13	8.75	Sulfur metabolism	L-homoserine-O-acetyltransferase,
MHT1	-3.06	8.34	Sulfur metabolism	S-methylmethionine-homocysteine methyltransferase
SPS4	-2.92	7.57	Unknown	Protein whose expression is induced during sporulation
SUL2	-2.90	7.46	Sulfur metabolism	High affinity sulfate permease
MMP1	-2.74	6.68	Sulfur metabolism	High-affinity S-methylmethionine permease,
YDR379C-A	-2.62	6.15	Unknown	Putative protein of unknown function
YLR053C	-2.57	5.94	Unknown	Putative protein of unknown function

PHO89	-2.52	5.74	Phosphate transport	Na+/Pi cotransporter,
MET32	-2.52	5.74	Sulfur metabolism	Zinc-finger DNA-binding transcription factor
COX7	-2.52	5.74	Electron transport chain	Subunit VII of cytochrome c oxidase
YFL051C	-2.44	5.43	Unknown	Putative protein of unknown function;
CYC1	-2.42	5.35	Electron transport chain	Cytochrome c, isoform 1
YGR153W	-2.38	5.21	Unknown	Putative protein of unknown function
EMC6	-2.26	4.79	Protein folding	Member of conserved ER transmembrane complex
MET10	-2.21	4.63	Sulfur metabolism	Subunit alpha of assimilatory sulfite reductase
HPF1	-2.21	4.63	cell wall organization and biosynthesis	Haze-protective mannoprotein
YLR460C	-2.19	4.56	Unknown	Member of the quinone oxidoreductase family,
CSN9	-2.16	4.47	mating pheromone response	Subunit of the Cop9 signalosome
PHM6	-2.15	4.44	Unknown	Protein of unknown function
MXR1	-2.12	4.35	Oxidative stress response	Methionine-S-sulfoxide reductase,
DAD4	-2.09	4.26	Chromosome segregation	Component of the DASH complex, a microtubule-binding subcomplex
GRX8	-2.09	4.26	Sulfur metabolism	Glutaredoxin that employs a dithiol mechanism of catalysis;
MIC14	-2.06	4.17	Unknown	Mitochondrial protein of unknown function
RAD59	-2.02	4.06	DNA amplification	Repair of double-strand breaks in DNA
MNN4	-1.98	3.94	Protein glycosylation	Putative regulator of mannosylphosphate transferase Mnn6p
LSM2	-1.87	3.66	mRNA splicing	Lsm protein; involved in mRNA decay
YGL230C	-1.87	3.66	Unknown	Putative protein of unknown function
EFG1	-1.83	3.56	RNA processing	Required for maturation of 18S rRNA
YDL085C-A	-1.83	3.56	Unknown	Putative protein of unknown function
MET17	-1.82	3.53	Sulfur metabolism	O-acetyl homoserine-O-acetyl serine sulfhydrylase
RSB1	-1.81	3.51	Fatty acid transport	Putative integral membrane transporter

CAR2	-1.77	3.41	Arginine catabolism	L-ornithine transaminase
AIM34	-1.75	3.36	Unknown	Protein of unknown function
CNL1	-1.75	3.36	Endosome organization	Component of the biogenesis of lysosome-related organelles complex-1 (BLOC-1)
DTD1	-1.74	3.34	tRNA metabolism	D-tyrosyl-tRNA(Tyr) deacylase
DYN2	-1.74	3.34	Cargo trafficking	Cytoplasmic light chain dynein
CMC2	-1.73	3.32	Mitochondrial respiration	Respiratory chain complex assembly or maintenance
AZR1	-1.71	3.27	Azole transport	Plasma membrane transporter of the MFS superfamily
YJR011C	-1.68	3.20	Unknown	Putative protein of unknown function
YNL122C	-1.68	3.20	Translation	Putative mitochondrial ribosomal protein
QCR10	-1.65	3.14	Mitochondrial electron transport	Cytochrome b-c1 complex subunit 10
SDC1	-1.64	3.12	Chromatin silencing at telomeres	Subunit of the COMPASS (Set1C) complex
CHZ1	-1.64	3.12	Chromatin remodeling	Histone chaperone for Htz1p/H2A-H2B dimer
YPL225W	-1.62	3.07	Unknown function	May interact with ribosomes,
IES5	-1.62	3.07	Telomere maintenance	Non-essential INO80 chromatin remodeling complex subunit
ALG13	-1.56	2.95	Oligosaccharide biosynthesis	Catalytic component of UDP-GlcNAc transferase
SPO16	-1.55	2.93	Meiotic recombination	Synaptonemal complex assembly
BLS1	-1.55	2.93	Endosome organization and transport	Biogenesis of lysosome-related organelles complex 1 subunit
YCR102C	-1.53	2.89	Unknown	Putative protein of unknown function
YLR456W	-1.52	2.87	Vitamin B6 metabolism	Pyridoxamine 5'-phosphate oxidase
YLR346C	-1.51	2.85	Unknown	Mitochondrial putative protein of unknown function
COX12	-1.50	2.83	Oxidative phosphorylation	Subunit VIb of cytochrome c oxidase
TFB5	-1.50	2.83	Transcriptional regulation	Component of the RNA polymerase II general transcription and DNA repair factor TFIIH

Table S5: **list of genes found to be down regulated in presence of cystine**

Gene Name	GmeanF	Fold Change	Pathway	Description
YOL079W	-1.87	3.66	Unknown	Dubious open reading frame
HAC1	-1.46	2.75	Unfolded protein response	Basic leucine zipper (bZIP) transcription factor
COX4	-1.39	2.62	Electron transport chain	Subunit IV of cytochrome c oxidase
OPT1	-1.16	2.23	Glutathione and phytochelatin transport	Proton-coupled oligopeptide transporter of the plasma membrane
LRO1	-1.14	2.20	Triglyceride biosynthesis	Acyltransferase that catalyzes diacylglycerol esterification
MET3	-1.10	2.14	Sulfur metabolism	ATP sulfurylase, catalyzes the primary step of intracellular sulfate activation
RSB1	-1.10	2.14	Unknown	Suppressor of sphingoid LCB sensitivity
MNN4	-1.04	2.06	Protein glycosylation	Putative positive regulator of mannosylphosphate transferase Mnn6p
NDE1	-1.04	2.06	Mitochondrial respiratory chain	Mitochondrial external NADH dehydrogenase,
MET2	-1.04	2.06	Sulfur metabolism	L-homoserine-O-acetyltransferase
CAR2	-0.99	1.99	Arginine biosynthesis	L-ornithine transaminase
SUL1	-0.99	1.99	Sulfur metabolism	High affinity sulfate permease
ERC1	-0.97	1.96	Unknown	Putative protein of unknown function
MET10	-0.96	1.95	Sulfur metabolism	Subunit alpha of assimilatory sulfite reductase
YPR089W	-0.96	1.95	Unknown	Protein of unknown function
SPS4	-0.96	1.95	Unknown	Protein whose expression is induced during sporulation
SUL2	-0.96	1.95	Sulfur metabolism	High affinity sulfate permease
SCT1	-0.95	1.93	Glycerolipid biosynthesis	Glycerol 3-phosphate/dihydroxyacetone phosphate
SPT5	-0.93	1.91	Transcriptional elongation	Protein involved in regulating Pol I and Pol II transcription
YMR086C-A	-0.92	1.89	Unknown	Dubious open reading frame
LSB6	-0.92	1.89	Actin polymerization	Type II phosphatidylinositol 4-kinase
YFR018C	-0.91	1.88	Unknown	Putative protein of unknown function
FET4	-0.91	1.88	Iron homeostasis	Low-affinity Fe(II) transporter of the

				plasma membrane
YGR054W	-0.89	1.85	Translation	Eukaryotic initiation factor (eIF) 2A
YVC1	-0.87	1.83	Cation homeostasis	Vacuolar cation channel
YBL070C	-0.86	1.82	Unknown	Dubious open reading frame
NOP58	-0.85	1.80	Pre RNA processing	involved in producing mature rRNAs and snoRNAs
YAL004W	-0.85	1.80	Unknown	Dubious open reading frame
YOR282W	-0.83	1.78	Unknown	Dubious open reading frame
YPR142C	-0.83	1.78	Unknown	Dubious open reading frame
MET6	-0.82	1.77	Sulfur metabolism	Cobalamin-independent methionine synthase
SSN2	-0.82	1.77	Transcriptional regulation	Subunit of the RNA polymerase II mediator complex
SSA2	-0.81	1.75	Protein folding	ATP binding protein involved in protein folding and vacuolar import of proteins
NCE102	-0.81	1.75	Unknown	Protein of unknown function
SYH1	-0.81	1.75	Unknown	unknown function
ATM1	-0.80	1.74	Iron sulfur biogenesis	Mitochondrial inner membrane ATP-binding cassette (ABC) transporter

Table S6 Common list of genes found to be up regulated in presence of cystine/cysteine.

Gene Name	Fold change cysteine	Fold change cystine	Description
ARG3	18.13	7.21	Ornithine carbamoyltransferase
FMP23	18	8.82	Putative protein of unknown function
TIS11	14.22	11.88	mRNA-binding protein : iron starvation
SRL4	10.85	3.48	Protein of unknown function
TMT1	9.45	4.76	Trans-aconitate methyltransferase
RTC2	9.06	4.29	YPQ3 vacuolar cationic amino acid transporter
ARG8	9.06	3.41	Acetylornithine aminotransferase,
ZRT1	8.75	3.07	High-affinity zinc transporter (Plasma membrane)
PKP2	8.22	3.2	Mitochondrial protein kinase
CPA1	8.22	3.18	Small subunit of carbamoyl phosphate synthetase
SSU1	8.06	2.79	Plasma membrane sulfite pump
FIT2	7.78	5.21	Mannoprotein : incorporated into the cell wall
ARG5,6	7.36	4.2	Acetylglutamate kinase and N-acetyl-gamma-glutamyl-phosphate reductase

YLR152C	6.54	3.66	Putative protein of unknown function
YGL117W	6.19	3.84	Putative protein of unknown function
SIT1	5.35	2.81	Ferrioxamine B transporter,
ARN1	4.56	3.63	recognizes siderophore-iron chelates
ORT1	4.56	3.12	Arginine biosynthesis
ARG4	4.2	2.64	Argininosuccinate lyase
NQM1	2.41	2.66	Transaldolase of unknown function

Table S7: Common list of genes found to be down regulated in presence of cystine/cysteine

**Gene Name**	**Fold change cysteine**	**Fold change cystine**	**Description**
MET3	14.03	2.14	ATP sulfurylase
SUL1	10.48	1.99	High affinity sulfate permease
MET2	8.75	2.06	L-homoserine-O-acetyltransferase
SPS4	7.57	1.95	Protein whose expression is induced during sporulation
SUL2	7.46	1.95	High affinity sulfate permease
MET10	4.63	1.95	Subunit alpha of assimilatory sulfite reductase
RSB1	3.51	2.14	Suppressor of sphingoid LCB sensitivity of an LCB-lyase mutation
CAR2	3.41	1.99	L-ornithine transaminase
OPT1	2.69	2.23	Proton-coupled oligopeptide transporter
NDE1	1.99	2.06	Mitochondrial external NADH dehydrogenase,
MNN4	1.82	2.06	Putative positive regulator of mannosylphosphate transferase Mnn6p

Table S8: list of primers used for the RT-PCR analysis

**Primer Name**	**Sequence**
ARG8_F	GGCCAGAGAATTGGGACTTT
ARG8_R	GCATCCATCCCTTCTTCGATTA
ARG5,6_F	GACGTAGCTGCTGGTGAATTAG
ARG5,6_R	CCCGTGGAGCCATTGATAATAC
RTC2_F	CTTGAGCTTGGCAGAACAATAG
RTC2_R	CGGCAATAAGTACACCTGAAAC
SSU1_F	TGGCTGCATGCCATTATCT
SSU1_R	CCTGTGTCATTGGTGGTATCT
TIS11_F	TCGGCAGTTTCATTCTCTCC
TIS11_R	GTAGTAGGTGCCGTGCTATTC
